# Medical and Surgical Directory of the United States

**Published:** 1886-08

**Authors:** 


### Reviews.

**Medical and Surgical Directory of the United States.** Complete in one volume. Price $7.00. R. L. Polk & Co., Publishers, Detroit, Mich. 1886.

There are very few of our readers who have not, during the past year, been made aware that the celebrated publishing house of Polk & Co. were engaged in getting out a work which, they promised, should contain the names of all the doctors in the United States. With some knowledge of the labor and expense which such an undertaking would involve, we had little expectation that the result would be satisfactory to the profession. We must confess, however, that we are most happily disappointed. The book, now before us, contains not only the names of 80,000 persons, arranged alphabetically, but also gives the list of practitioners arranged by States, with post-office address; date of graduation; together with name of college, or other licensing body; and also a list of all the medical colleges in the United States and Canada; the medical journals; medical societies; a synopsis of the laws relating to the profession in each State; an official list of the officers of the medical departments of the United States Army, Navy and Marine Service, etc.

We have taken some pains to test the correctness of the directory by comparing it with our address list, and we are surprised at the few errors and omissions we have been able to find. That such a volume can be sold at the low price of seven dollars, is astonishing. Through this directory, any one will be able to find the present location and address of any physician whose name he knows.

